# A mechanistic study on the inhibition of bacterial growth and inflammation by *Nerium oleander* extract with comprehensive in vivo safety profile

**DOI:** 10.1186/s12906-021-03308-z

**Published:** 2021-05-01

**Authors:** Yousra Shafiq, Syed Baqir Shyum Naqvi, Ghazala H. Rizwani, Muhammad Arif Asghar, Rabia Bushra, Sana Ghayas, Ahad Abdul Rehman, Muhammad Asif Asghar

**Affiliations:** 1 Department of Pharmaceutics, Institute of Pharmaceutical Sciences, Jinnah Sindh Medical University, Rafiqui H. J Shaheed Road, Karachi, 75510 Pakistan; 2 Department of Pharmaceutics, Faculty of Pharmacy, Hamdard University, Karachi, Pakistan; 3 Research Department, Faculty of Eastern Medicine, Hamdard University, Karachi, Pakistan; 4 Department of Pharmaceutics, Dow College of Pharmacy, Dow University of Health Sciences, Karachi, Pakistan; 5 Department of Pharmacology, Institute of Pharmaceutical Sciences, Jinnah Sindh Medical University, Karachi, Pakistan; 6 Food and Feed Safety Laboratory, Food and Marine Resources Research Centre, PCSIR Laboratories Complex, Shahrah-e-Salimuzzaman Siddiqui, Off University Road, 74200, Karachi, Sindh 75280 Pakistan

**Keywords:** *Nerium oleander*, Time killing kinetics, Anti-inflammatory activity, Acute and sub-acute toxicity

## Abstract

**Background:**

*Nerium oleander* (L.) is well known traditionally used medicinal plant with several pharmacological activities. However, the anti-bacterial, anti-inflammatory activity and in vivo toxicity potential of floral parts of this plant are not reported. Therefore the present study was designed to investigate these activities of *Nerium oleander* ethanolic flower extract (NOEE) in different animal models.

**Methods:**

Antimicrobial activity of plant extract was compared with five different antibiotics using the disk diffusion method. The time-killing kinetic assay and bacterial killing mechanism of NOEE were also performed. Anti-inflammatory activity was assessed using granuloma induced by cotton-pellet, rat paw edema induced by carrageenan and levels of different inflammatory biomarkers on healthy Wistar rats. The protein and mRNA expressions of nitric oxide (NO), prostaglandin E_2_ (PGE_2_), tumor necrosis factor-α (TNF-α) and interleukin-1β (IL-1β) were also measured. Acute (14 days) and sub-acute (28 days) oral toxicity studies were also performed on healthy Sprague Dawley rats.

**Results:**

NOEE produced highly significant (*P < 0.005*) and significant (*P < 0.05*) zones of inhibition at 30 mg/mL and 20 mg/mL respectively against most of the tested bacterial strains. NOEE produced a more drop in viable counts of Gram-negative isolates within 20 min. After 12 h exposure with NOEE, the SEM images of *MRSA* showed the destruction of cell membrane. NOEE showed highly significant *(P < 0.005)* anti-inflammatory activity in cotton-pellet and carrageenan inflammatory models. In addition, treatment with NOEE also decreased the production of NO, PGE_2_, TNF-α and IL-1β in the rat paw after treated with carrageenan. Similarly, NOEE also suppressed the inducible nitric oxide synthase (iNOS), TNF-α, IL-1β, and cyclooxygenase-2 (COX-2) mRNA expressions. It is also showed highly significant reduction in total leukocyte count (73.09%) and C-reactive protein levels (54.60%). NOEE also inhibited COX-1, COX-2, 5-LO and 12-LO in a highly significant manner. Moreover, acute and sub-acute toxicity studies of NOEE in rats confirm the toxicity with hepatotoxicity at higher doses (2000 mg/kg) i.e. four times greater than the therapeutic dose.

**Conclusion:**

It is concluded that crude flower extract of *N. oleander* is a potent antimicrobial and anti-inflammatory agent with no toxicity potential at therapeutic doses.

**Supplementary Information:**

The online version contains supplementary material available at 10.1186/s12906-021-03308-z.

## Background

In order to balance the host defense against pathogenic threats, an inflammatory response is required. Though, uncontrollable eicosanoid production is usually associated with multiple chronic inflammatory diseases [1]. It has been reported in different studies that mostly inflammation is initiated by the injury of living cells either by the outbreak of microorganisms, poor or weak immune response and sometimes by physical agents [2]. However, the fundamental response of inflammation is considered to be a protective response by eradicating the initial cause of cell injury, following the start of the cell repair process [3]. The numbers of anti-inflammatory mediators are responsible for inflammation resolution along with monocyte recruitment for the deletion of tissue debris or cell. Chronic inflammation usually causes burden in terms of pathological conditions in both developing and under-developing countries [4].

There are numbers of drugs available in the market to treat and control inflammatory distress including NSAIDs, steroids and immune suppressants while such medications are also associated with multiple adverse effects [5]. So, the present approach of study is to investigate a drug candidate with minimum side effects and maximum efficacy from the green world. In recent years, various researchers have focused on natural products that are derived from medicinal plants i.e. flavonoids, alkaloids, polyphenols, steroids, coumarins and terpenes because of their broad range of pharmacological importance [6–9]. Recently, the World Health Organization (WHO) reported that around 80% of the human population utilized herbal medicines for the management of some aspects of primary health care [10].

**Fig. 1:**
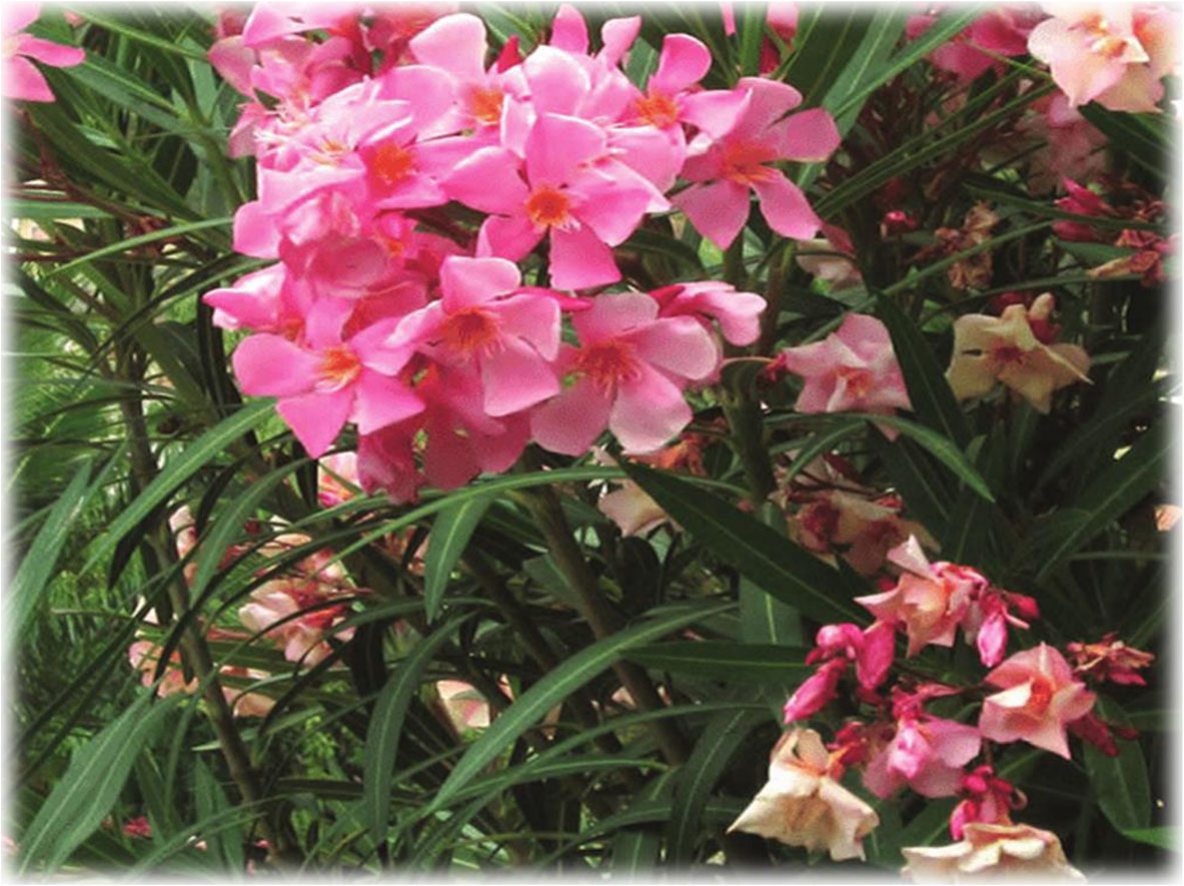
Plant of *Nerium oleander* with its flowers

*Nerium oleander* belongs to the family *Apocynaceae*, is an evergreen shrub and is found widely distributed throughout the world [11]. The image of the plant along with its flowers is also given in Fig. 1**.** Plant was reported for its antibacterial [12], antifungal [13], antidiabetic [14], antioxidant [15], antitumor [16] and hepatoprotective activities [17]. Phyto-constituents such as flavonoids, triterpenes, anthraquinones, coumarins, cardiac glycosides like oleandrin and nerine have been reported in this plant [18].

It has been reported that plants containing a high proportion of flavonoids and saponins showed good analgesic and anti-inflammatory potentials while ethanolic extract of *N. oleander* also possessed a rich quantity of such phytochemical constituents responsible for these activities [6, 19]. Moreover, various plants of the *Apocynaceae* family were evaluated for their anti-inflammatory activity in different animal models. Fruits extract of *Hancornia speciosa* showed remarkable anti-inflammatory activity against inflammatory induced animal models [20]. Saleem et al. evaluated the anti-inflammatory activity of *Gendarussa vulgaris* leaves against carrageenan-induced paw edema [21]. In addition, very few studies extract out regarding the anti-inflammatory activities of *N. oleander* with few inflammatory induced models.

Even though, the flowers of this plant are traditionally used topically in the treatment of inflammation due to bacterial infections particularly in rural areas of Pakistan. However, there is no scientific data reported on the comprehensive in vivo anti-inflammatory and anti-bacterial activity of *N. oleander* flowers. Also, investigation of the safety profile of this plant is highly required given the pervasive use. Thus, the present study was performed to assess the time killing antibacterial assay, anti-inflammatory and toxicity activities of *N. oleander* flowers extract at different doses in animal models.

## Methods

### Collection and extraction of plant material

Fresh flowers of *Nerium oleander* were purchased from a local herb research market of sadder town in Karachi-Pakistan in the month of June, a peak time for their growth. It was identified by the meritorious Professor and Pharmacognosist Prof. Dr. Ghazala H. Rizwani at Center of Plant Conversation, University of Karachi, Pakistan and the plant voucher specimen was deposited at the Center of Plant Conversation, University of Karachi, Pakistan with voucher specimen no. CE–0091. The collected flowers were washed, shade dried and course powdered by an electric grinder. The amount of 500 g dried powdered plant material was extracted using Soxhlet extractor with 1 L of 50% ethanol for 24 h. Plant material was filtered and concentrated under reduced pressure using a rotary vacuum evaporator at 40 °C (IKA, Germany) [6]. The final product of *Nerium oleander* ethanolic extract (NOEE) was a blackish brown color mass with a 7.56% yield.

### Phytochemical screening

Different phytochemical screening tests were performed in order to determine the phytochemical constituents in NOEE. Total flavonoids and cardiac glycosides in plant extract were estimated using Shinoda and Keller-Kiliani tests. Total phenolic contents in the extract were assessed using the ferric chloride test, whereas total alkaloids were determined using Mayer’s reagent. Borntrager’s and froth tests were used for the estimation of total anthraquinones and saponins levels respectively while total phytosterols were estimated using Liebermann–Burchard test [22].

### Antibacterial susceptibility testing’s

#### Determination of zones of inhibitions (ZIs)

Five different antibiotic discs of 10 μg/disc concentrations were used as standard, namely amoxicillin, erythromycin, gentamycin, levofloxacin and tetracycline (OXOID, UK). Different clinical isolates including *Bacillus subtilis* (LT 0412), *Escherichia coli* (LT 0613), *Klebsiella pneumonia* (LT 0843), *Pseudomonas aeruginosa* (LT 0618), Methicillin-resistant *Staphylococcus aureus (MRSA) (LT 0251), Staphylococcus aureus* (LT 0871), *Staphylococcus epidermidis* (LT 0482), *Salmonella enterica* (LT 0305), *Streptococcus fecalis* (LT 0711), *Streptococcus pyogenes* (LT 0121) and *Salmonella typhi* (LT 0235) were obtained and identified by Pathologist from the pathological laboratory of Darul-Sehat Hospital Karachi Pakistan. These bacterial strains reported a highly resistant pattern in previous studies, particularly in Asian countries. Plant discs with 20 μL extract solution (6 mm in diameter) of different concentrations, i.e. 10, 15, 20 and 30 mg/mL were used for the evaluation of its antibacterial activity using a Clinical Laboratory and Standard Institute (CLSI) approved disc diffusion method [23, 24]. The bacterial culture was inoculated in sterile Muller Hinton agar (MHA) (Oxoid, Hemisphere, England) and incubated for 24 h at 37 °C. The 0.5 McFarland standard was used to achieve 10^6^ cell/mL concentrations of each strain. Antibacterial discs were placed on the MHA plates and incubated for 24 h in an incubator (Thermo Fisher Scientific, USA) at 37 °C. Inhibition zones were measured using a digital Vernier caliper.

#### Determination of minimum inhibitory concentration (MIC) and minimum bactericidal concentration (MBC)

MICs and MBCs of NOEE against tested isolates were determined using the broth dilution method [25]. Briefly, the plant extract with a concentration of 50 mg/mL was prepared in a nutrient broth and make several dilutions up to 0.1 mg/mL using the serial dilution method. All tested bacterial strains were adjusted to the concentration of 1 × 10^6^ cfu/mL as McFarland standard. After overnight incubation of inoculated microbial plates, optical densities (ODs) were measured using an ELISA reader (Infinite 200; USA) at 600 nm. In addition, MBCs of plant extract was determined by spreading the solution of microbial strains on nutrient agar plates and incubated at 37 °C for 24 h. After an incubation period, bacterial colonies on each inoculated plate were observed [26, 27]. Each experiment was performed three times and mean ± SD values of each test were reported.

#### Killing kinetics assay

The time-killing kinetics study on NOEE was performed according to the method defined in our previous study [28]*.* The bacterial cells were grown in logarithmic phase up to 8 logs cfu/mL in nutrient broth then treated with plant extract in the concentration equal to obtained MBCs of each tested bacterial isolate. Bacterial culture was drawn after every 5 min and plated on Tyramide Signal Amplification (TSA) in order to measure the bacterial cell viability. The time-killing kinetic plot was constructed between the viability of bacterial isolates in Log cfu/mL and time in min.

#### Effects of NOEE on bacterial cells morphology

The morphological study of bacterial cells after exposure with NOEE at their MIC was performed according to the method described in our previous study using a scanning electron microscope (SEM) (Joel, Model no SEM-2364, Japan) [29]. SEM study was carried out on *MRSA* cells since it is the most common virulence bacteria for human and cause several infections including urinary tract infections, wounds infections and different systemic infections. Before and after 6 and 12 h treatment with NOEE, bacterial suspension was placed on filter membrane and dried for 2 h. Then the bacterial culture was fixed with the solution of glutaraldehyde in 2.5% phosphate buffer. The bacterial isolate was stained for 60 min with OsO_4_ solution in 1% phosphate buffer and dehydrated with a hydroalcoholic mixture. Finally, the gold-coated filter membrane was analyzed.

### Evaluation of anti-inflammatory activity

#### Animals

Sprague Dawley rats of both sexes were obtained from the animal house of the Pakistan Council of Scientific and Industrial Research (PCSIR), with an average body weight of 220–250 g. The filled consent form was submitted to the animal house in-charge. During the whole study, both control and tested rats were kept in plastic cages with a controlled temperature of 23 °C ± 2 °C and humidity of 50–60% in a 12 h light-dark cycle. Animal handling was performed according to the care and use of Laboratory Animals guidelines provided by the National Institute of Health (NIH). Moreover, the study was approved by a local departmental animal ethical committee of Pakistan Council of Scientific and Industrial Research (PCSIR), Karachi-Pakistan with the approval number of 05/04/KK/PCSIR. At the end of the study, the animals were sacrificed using the cervical dislocation method with all possible effort being given to minimize suffering. Prior to euthanasia, each animal was sedated with an intravenous injection of medetomidine in the concentration of 2 μg/kg [30].

#### Cotton pellet induced granuloma in rats

Animals were divided into five groups and each group was composed of 10 rats. Three test groups were received NOEE in the concentrations of 125, 250 and 500 mg/kg, standard group was taken 10 mg/kg of indomethacin (Epoch Pharmaceutical Ltd. Pakistan) while distilled water was administered to the control group. After 1st dose of administration, approximately 10 mg of the aseptic cotton pellet was implanted subcutaneously in the back area of the anesthetized animals and treated once daily with standard and test solutions for 7 days. On the 8th day, animals were killed and implanted cotton pellets were removed and dried using a hot air oven at 65 °C. Then, weighed the pellets and calculate the weight gained after implantation, which was considered as granuloma formation [31].

The percent inhibition (PI) of granuloma formation in treated animals was calculated in comparison with a control group using the following formula:

$$ \mathrm{PI}=\frac{\mathbf{Weight}\ \mathbf{of}\ \mathbf{cotton}\ \mathbf{pellet}\ \mathbf{in}\ \mathbf{control}\ \mathbf{group}-\mathbf{Weight}\ \mathbf{of}\ \mathbf{cotton}\ \mathbf{pellet}\ \mathbf{in}\ \mathbf{treated}\ \mathbf{group}\ }{\mathbf{Weight}\ \mathbf{of}\ \mathbf{cotton}\ \mathbf{pellet}\ \mathbf{in}\ \mathbf{control}\ \mathbf{group}} \times 100 $$

#### Carrageenan-induced edema in the rat paw

Six groups of animals were used in which 20 mg/kg of diclofenac sodium (Barrett Hodgson Ltd., Pakistan) was given to the standard group, Naive group had not received any type of treatment or carrageenan while the remaining groups were received the same as described in cotton pellet induced granuloma study. After 1st dose administration, inflammation was induced by given 0.1 mL (1%) carrageenan (Kasei chemical industry, Japan) in the right hind paw. Plethysmometer was used to measure the edema in the rat paw at initial and after various times-intervals. Then, the actual volume of edema was measured by subtracting the initial and subsequent readings [32]. The percent inhibition (PI) of edema formation in treated animals was calculated using the following formula:

$$ \mathrm{PI}=\frac{\mathbf{Mean}\ \mathbf{volume}\ \mathbf{of}\ \mathbf{edema}\ \mathbf{in}\ \mathbf{control}\ \mathbf{group}-\mathbf{Mean}\ \mathbf{volume}\ \mathbf{of}\ \mathbf{edema}\ \mathbf{in}\ \mathbf{treated}\ \mathbf{group}\ }{\mathbf{Mean}\ \mathbf{volume}\ \mathbf{of}\ \mathbf{edema}\ \mathbf{in}\ \mathbf{control}\ \mathbf{group}}\times 100 $$

#### Sample preparation of rat paw tissue

Rats were sacrificed using above defined method after treatment with carrageenan and the paw was harvested immediately. Then the paw was washed with sterile cold saline and stored at − 90 °C till further use. A paw homogenate (10% w/v) was prepared for biochemical analysis using the 0.1 M sodium phosphate buffer having a pH of 7.4 with a protease inhibitor cocktail. The prepared homogenate was centrifuged for 15 min at 9000×g and 4 °C. The supernatants were drawn and then stored at − 90 °C until use [33].

#### Nitrite oxide (NO) assay

The concentration of nitrite in the supernatant indicated the production of NO and it was measured using the Griess reaction method [34]. A volume of 100 μL supernatant was mixed with the equal volume of Griess reagent which containing 50 μL aqueous N-1-naphthylethylenediamine dihydrochloride (1%) and 50 μL sulfanilamide (1%) in phosphoric acid (5%). Before the sample absorbance was measured at the wavelength of 540 nm using a microplate reader (BMG, Germany), each sample was incubated at room temperature for 10 min. The amount of nitrite content was given in percentage.

#### PGE_2_ assay

A commercially available kit (R&D Systems, UK) was utilized for the determination of PGE_2_ level in the supernatant according to the instructions given by the manufacturer. Briefly, a volume of 50 μL supernatant was added in a 96-well plate coated with enzyme-linked PGE_2_ specific polyclonal antibodies and left for 20 h. After the reaction time, the free antibody-enzyme reagent was removed and the color intensity was measured after the addition of a substrate solution at 450 nm. The PGE_2_ content was given in percentage.

#### Cytokine determination

A commercially available kit (R&D Systems, UK) was also used to determine the TNF-α and IL-1β levels in the supernatant as per manufacturer instructions. A volume of 100 μL supernatant was added in a 96-well plate coated with enzyme-linked rat TNF-α and IL-1β specific polyclonal antibodies and left for 2 h reaction time. A reaction sample was washed to remove the free reagent of the antibody-enzyme complex. The color intensity was measured after the addition of a substrate solution at 450 nm. The TNF-α and IL-1β levels were given in percentage.

#### Real-time polymerase chain reaction (PCR) analysis

A total amount of RNA was isolated from the treated paw using TRIzolR reagent (Gibco, USA). Absorbance was measured at 260 nm for the calculation of extracted RNA concentrations. However, 260 and 280 nm absorbance ratio was used for the assessment of RNA quality and 1.9 to 2.1 were the acceptable values of A260/A280. An amount of 1.5 μg total RNA was used for the synthesis of cDNA using the cDNA reverse transcription kit (Applied Biosystems, USA). However, PCR analysis was performed using PCR TaqMan master kit (2×) (Applied Biosystems, USA) and TaqMan mouse gene expression assays (Applied Biosystems, assay ID: COX-2, Rn01483827_g1; iNOS, Rn00561646_m1; IL-1β, Rn00434228_m1; TNF-α, Rn02061804_s1 and β-actin, Rn00667869_m1). The reaction times were 2 and 10 min at 50 °C and 95 °C respectively then run 60 cycles for 15 s at 95 °C and 1 min at 60 °C on a Real-Time PCR system (A453, Applied Biosystems, USA) while data analysis was made using sequence detection software version 2.0 (Applied Biosystems, Inc., USA). The mRNA (TNF-α, IL-1β, COX-2 and iNOS) relative expressions were normalized with the β-actin amount in the same cDNA according to the relative 2^–ΔΔCT^ quantification method [35]. The fold alteration in the cDNA target gene was determined relative to the β-actin control using the formula mentioned below:

Fold alteration in cDNA = 2^−ΔΔCT^

Where,

$$ \Delta \Delta \mathrm{CT}=\left({\mathrm{Ct}}_{\mathrm{target}\ \mathrm{gene}}-{\mathrm{Ct}}_{\beta -\mathrm{actin}}\right)-\left({\mathrm{Ct}}_{\mathrm{control}}-{\mathrm{Ct}}_{\beta -\mathrm{actin}}\right) $$

#### Determination of inflammatory biomarkers

##### Determination of leukocytes and C-reactive protein (CRP) levels

There are similar 6 groups of rats were used as defined in the carrageenan-induced edema model except for the standard group that was received 100 mg/kg of acetylsalicylic acid (Atco Laboratories Ltd. Pakistan). After a 3 h administration of 0.05 N acetic acid (Sigma-Aldrich, USA), total leukocyte count and CRP levels were measured in peritoneal exudate [36].

##### Cyclooxygenase-1 and cyclooxygenase-2 assay

The inhibitory potential of NOEE on COX-1 and COX-2 was evaluated according to the method reported by Li et al. in 2003 [37]. Briefly, each enzyme was activated by the standard activation method then NOEE and indomethacin (standard) were added individually in each activated enzyme in the concentrations of 1–100 μg/mL and 10 μg/mL respectively. Arachidonic acid (Rofarma-Italia, Italy) was added to start the reaction. The samples were incubated for 10–15 min at 37 °C and then terminated the reaction by adding 4 M formic acid. The metabolites of arachidonic acid (prepared during this reaction) were separated and analyzed with the help of a liquid scintillation counter.

##### 5-Lipoxygenase and 12-Lipoxygenase assay

The standard and test solutions were treated with the same concentrations used in COX-1 and COX-2 assay with 5-lipoxygenase and 12-lipoxygenase individually for 10–15 min at 24 °C. Then arachidonic acid was added to start the enzymatic reactions. The 4 M formic acid was used to acidify the reaction. 5-Hydroxyeicosatetraenoic acid (5-HETE) and 12-HETE were obtained as an end product in 5-lipoxygenase and 12-lipoxygenase enzymatic reactions respectively. The quantities of these by-products were determined using a liquid scintillation counter [37].

### In vivo acute and sub- toxicity studies

#### Animals dosing

Healthy Sprague Dawley rats (10–12 weeks old) of both genders were divided into four groups, with 10 rats in each group. Distilled water was administered to the control group; three groups received 500 mg/kg, 1000 mg/kg and 2000 mg/kg NOEE once daily orally individually for 28 days. The extract doses for testing were adopted based on a preliminary acute toxicity study on plant extract where a lethal dose (LD_50_) was found to be > 5000 mg/kg. The OECD guidelines for toxicity studies were followed for this study [38].

#### Clinical examinations, body weight and relative organ weights

After dosing of NOEE, any sign of toxicity was recorded multiple times a day. Plant extract effects were observed on animal general health, behavior, skin and hairs. Initially and after 28 days dosing periods, bodyweight of each rat was noted. In addition, the weight of vital organs was measured and their relative organ weights were calculated on the basis of the total body weight of each rat.

#### Hematological and biochemical analysis

Both control and treated rats were sacrificed after the last dosing, 5 mL blood sample was drawn from the femoral artery of each rat and added in 20 mg/mL of EDTA (anticoagulant) containing tube for hematological and other biochemical analysis. Blood samples were analyzed to evaluate the change in the number of erythrocytes (RBCs) and white blood cells (WBCs) using an automated blood sample analyzer (Beckman Coulter, U.S). In addition, serum electrolytes, erythrocyte sedimentation rate (ESR) and hemoglobin levels were also estimated. Biochemical analysis was also performed related to the enzymatic levels of the kidney and liver using an auto-analyzer (3400–230, Hitachi, Japan) [39].

### Statistical analysis

All results are given as their mean ± standard deviation. The antibacterial activity, anti-inflammatory activity and in vivo toxicity findings were subjected to analysis of variance (ANOVA), and post-hoc Tukey tests using SPSS statistical software (version 23). *P* < 0.05 and *P* < 0.005 were considered as the statistical significance and highly statistically significant results respectively. In addition, correlation coefficient and regression analysis were used to determine the relationship among time killing kinetics of different bacterial strains and cell viability respectively.

## Results

### Phytochemical screening of NOEE

The ethanolic fraction of *N. oleander* flowers was used for the determination of different phytochemical constituents and their percentage composition. Several phytochemical constituents were found in NOEE in different percentage crude yields such as cardiac glycosides (4.24%), flavonoids (4.21%), steroids (3.89%), terpenes (3.76%) alkaloids (3.21%), saponins (2.41%), phenols (1.92%) and tannins (0.11%) while anthraquinones levels were found in the lowest percentage composition i.e. 0.09%.

### Antibacterial susceptibility testing’s

#### Zone of inhibitions, MIC and MBC values

The antibacterial activities of different antibiotics and NOEE at different concentrations are presented in Table 1. Among all highly resistant clinical tested isolates; *MRSA, S. epidermidis, S. aureus and S. enterica* were highly susceptible to the plant extract with the highly significant *(P < 0.005)* ZIs were found i.e. 28.8 ± 5.74, 28.1 ± 5.44, 27.8 ± 6.92 and 27.7 ± 4.36 respectively at the concentration of 30 mg/mL. The values of MIC/MBC showed that NOEE has strong bacterial growth inhibitory and bactericidal activity against *S. aureus* (5.0/5.5 mg/mL)*, B. subtilis* (6.5/7.0 mg/mL), *MRSA* (8.0/8.0 mg/mL) and *E. coli* (8.0/9.0 mg/mL) as given in Table S1.

**Table 1:** Antimicrobial activity of crude ethanolic flowers extract of *N. oleander* and different standard antibiotics against clinical isolates at different concentrations

Clinical isolates	Zone of inhibitions in mm									
	NOEE				Antibiotics					Control (Water)
	10 mg/mL	15 mg/mL	20 mg/mL	30 mg/mL	Amoxicillin	Erythromycin	Gentamycin	Levofloxacin	Tetracycline	(100 μL/well)
***B. subtilis***	18.2 ± 3.74*	20.3 ± 4.80*	22.1 ± 3.75*	26.1 ± 4.05**	6.5 ± 0.24	20.4 ± 2.45**	7.5 ± 1.55	9.2 ± 2.63	14.1 ± 2.12*	0.00 ± 0.00
***E. coli***	14.0 ± 3.12	18.2 ± 3.18*	22.5 ± 5.11*	25.2 ± 3.44**	13.4 ± 3.28	17.5 ± 4.50*	14.2 ± 3.25*	13.2 ± 2.52	11.5 ± 2.85	0.00 ± 0.00
***K. pneumonia***	11.5 ± 2.16	12.4 ± 2.45	15.2 ± 3.31*	17.6 ± 3.70*	5.8 ± 1.42	18.2 ± 4.28*	12.2 ± 2.60	11.2 ± 2.57	10.4 ± 2.44	0.00 ± 0.00
***P. aeruginosa***	15.1 ± 3.37*	18.1 ± 5.91*	19.7 ± 3.70*	22.6 ± 3.75*	7.0 ± 1.37	17.2 ± 4.78*	10.2 ± 2.26	18.0 ± 4.63*	22.5 ± 5.19*	0.00 ± 0.00
***MRSA***	14.4 ± 3.41	18.7 ± 4.24*	23.7 ± 6.74**	28.8 ± 5.74**	5.6 ± 1.44	10.4 ± 2.75	6.2 ± 1.71	12.5 ± 2.14*	11.8 ± 2.72	0.00 ± 0.00
***S. aureus***	18.1 ± 4.94*	22.2 ± 4.16*	23.6 ± 3.52**	27.8 ± 6.92**	8.5 ± 1.74	17.5 ± 4.15*	9.6 ± 2.11	18.2 ± 3.21*	19.2 ± 5.71*	0.00 ± 0.00
***S. epidermidis***	14.3 ± 3.74*	19.5 ± 4.84*	21.4 ± 3.54*	28.1 ± 5.44**	8.2 ± 2.85	24.0 ± 4.24**	15.2 ± 3.73*	19.5 ± 4.64*	14.2 ± 3.02*	0.00 ± 0.00
***S. enterica***	15.1 ± 3.32*	19.0 ± 3.53*	23.0 ± 4.25**	27.7 ± 4.36**	6.5 ± 1.87	11.2 ± 2.74	8.3 ± 1.50	24.1 ± 4.32**	9.0 ± 2.27	0.00 ± 0.00
***S. fecalis***	6.6 ± 3.71	13.9 ± 2.92	15.3 ± 3.47*	17.2 ± 3.29*	7.2 ± 1.74	19.6 ± 4.03*	18.7 ± 4.21*	18.0 ± 4.56*	19.2 ± 3.57*	0.00 ± 0.00
***S. pyogenes***	13.1 ± 3.32	16.2 ± 4.42*	21.0 ± 3.63**	23.9 ± 3.90**	8.0 ± 2.87	19.0 ± 3.54*	14.2 ± 3.26*	18.7 ± 3.38*	17.5 ± 4.24*	0.00 ± 0.00
***S. typhi***	10.2 ± 2.16	12.9 ± 2.75	15.1 ± 2.92*	21.1 ± 4.11**	5.2 ± 0.69	11.4 ± 3.24	13.2 ± 3.82*	23.4 ± 4.13**	9.5 ± 2.62	0.00 ± 0.00

Results are mean ± S. D of triplicate experiments

*p ≤ 0.05 significant as compared to control, **p ≤ 0.005 highly significant as compared to control

#### Antibacterial killing kinetics

**Fig. 2:**
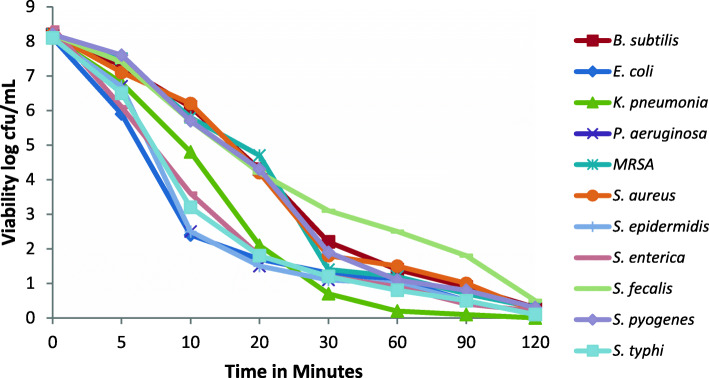
Growth profile of different isolates at different time interval after exposure to ethanolic extract of *Nerium oleander* at MBC of each microbe. All experiments were performed in triplicates. Linear relationship (R2 = 0.680) among viable cells counts of different bacterial strains at different time intervals while the viable cells counts of all bacterial strains significantly (R2 = - 0.876) decreases with increasing exposure time of NOEE

The growth profiles of all tested isolates with respect to time after exposure with the NOEE at MIC of each strain are shown in Fig. 2. This killing kinetics study was carried out for 120 min. NOEE produced a more drop in viable counts of *S. epidermidis, P. aeruginosa, E. coli, S. enterica* and *S. typhi* cells within 20 min. In addition, it was also observed that after exposure to NOEE, all tested isolates showed a stationary phase in bacterial growth after 2 h.

#### Bacterial killing mechanism

**Fig. 3:**
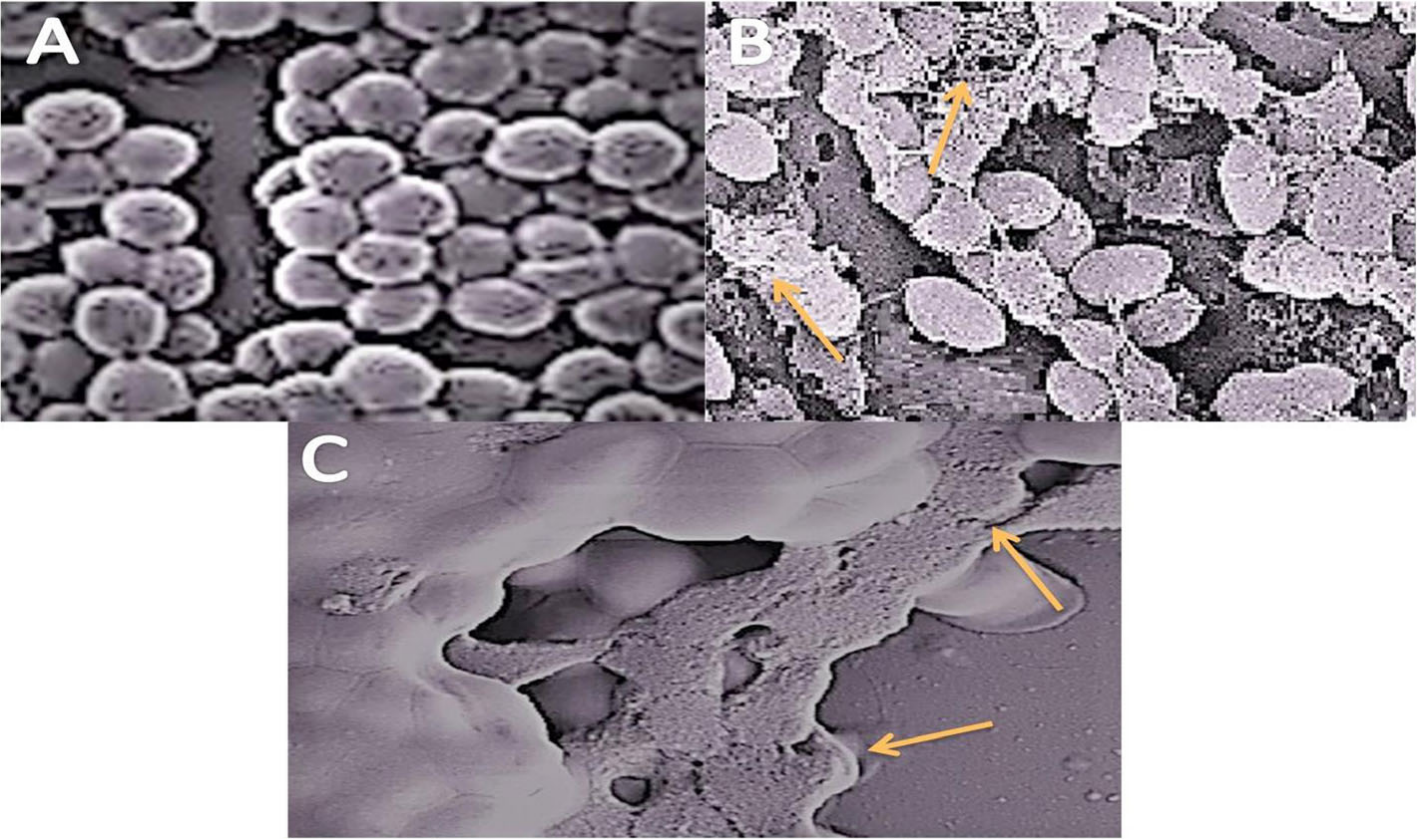
Morphology of *MRSA* at initial stage **a**, after 6 h **b**, and 12 h **c**, exposed to NOEE at their MIC

Figure 3 (a-c) displays the morphology of bacterial cells at the initial stage, then after 6 and 12 h of treatment with NOEE. Figure 3 a shows the normal morphology of *MRSA* cells at the initial stage of exposure to NOEE while after 6 h the cells were started to shrink as indicated in Fig. 3 b**.** However, Fig. 3**c** showed that after 12 h treatment with NOEE, the bacterial cell membrane was completely raptured resulting in the non-viability of *MRSA* cells

#### Anti-inflammatory activity

Overall animal health evaluations such as average weight variation, skin ulceration, loss of activity, diarrhea, hematuria, salivation, tremor, vomiting, edema and aggressive behavior were observed in all control and test groups before and during the total period of the experiment.

#### Cotton pellet induced granuloma in rats

**Fig. 4:**
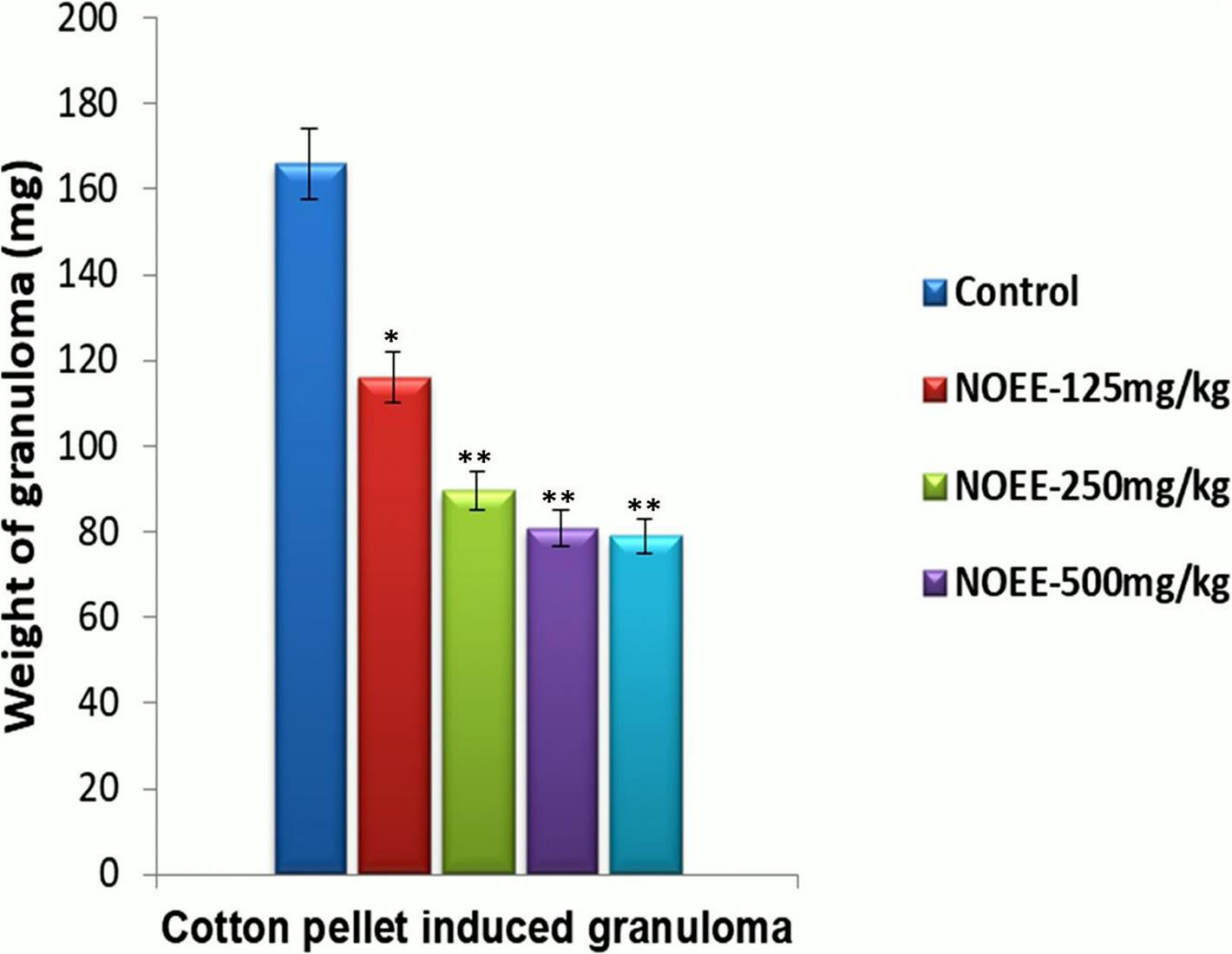
Effect of the NOEE on cotton pellet induced granuloma in Sprague dawley rats. Values are given as x̄ ± S.E.M. (*n* = 10), *p ≤ 0.05 and **p ≤ 0.005 significant and highly significant as compared to control

The results of the anti-inflammatory activity of NOEE on granuloma formation induced by a cotton pellet in rats are given in Fig. 4. It was observed that the anti-inflammatory effect of NOEE was found to be dose dependent. At 250 and 500 mg/kg doses of NOEE, a highly significant reduction in inflammation was observed compared with the control (*P < 0.005*). The standard indomethacin at 10 mg/kg produced very comparable activity with plant extract at 500 mg/kg (52.49% vs. 51.29%).

#### Carrageenan-induced edema in the rat paw

**Fig. 5:**
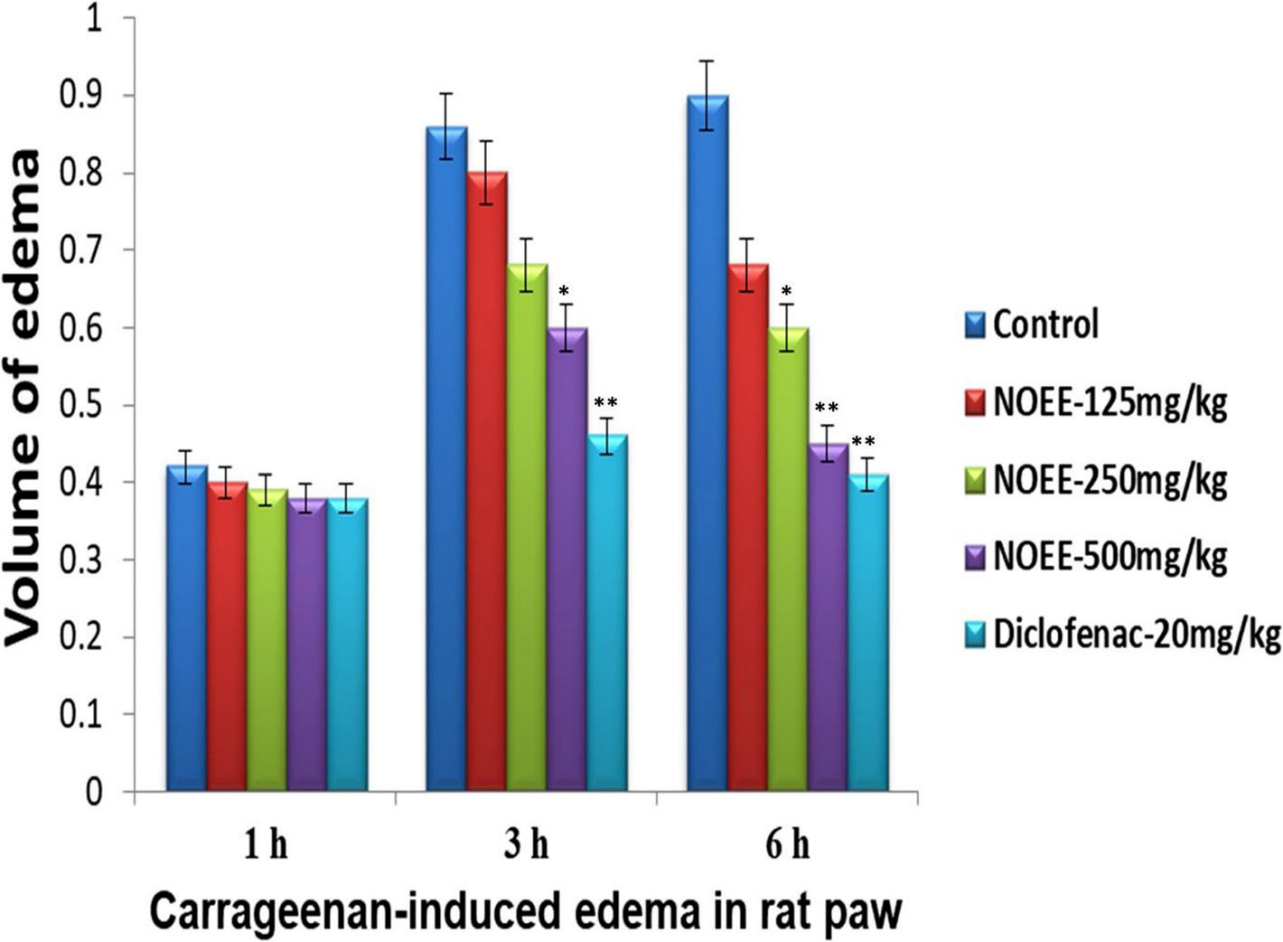
Effect of the NOEE on carrageenan-induced edema in rat paw; Values are given as x̄ ± S.E.M. (*n* = 10), *p ≤ 0.05 and **p ≤ 0.005 significant and highly significant as compared to control. The naïve group did not show any sign of edema (Naïve = 0.00)

The effects of different concentrations of NOEE on carrageenan-induced edema in rat paw are presented in Fig. 5. The anti-inflammatory activity of NOEE was dose dependent from the 3rd to 6th h. The significant (*P < 0.05*) and highly significant (*P < 0.005*) inhibitory effects were observed against carrageenan-induced inflammation at both doses of NOEE with the inhibition percentages of 37.77 and 50.00% respectively after 6 h. The anti-inflammatory effect of a standard drug (54.44%) was comparable with the tested plant extract.

#### Effects of NOEE on the production of NO and iNOS mRNA expression in carrageenan-induced rat paw edema

**Fig. 6:**
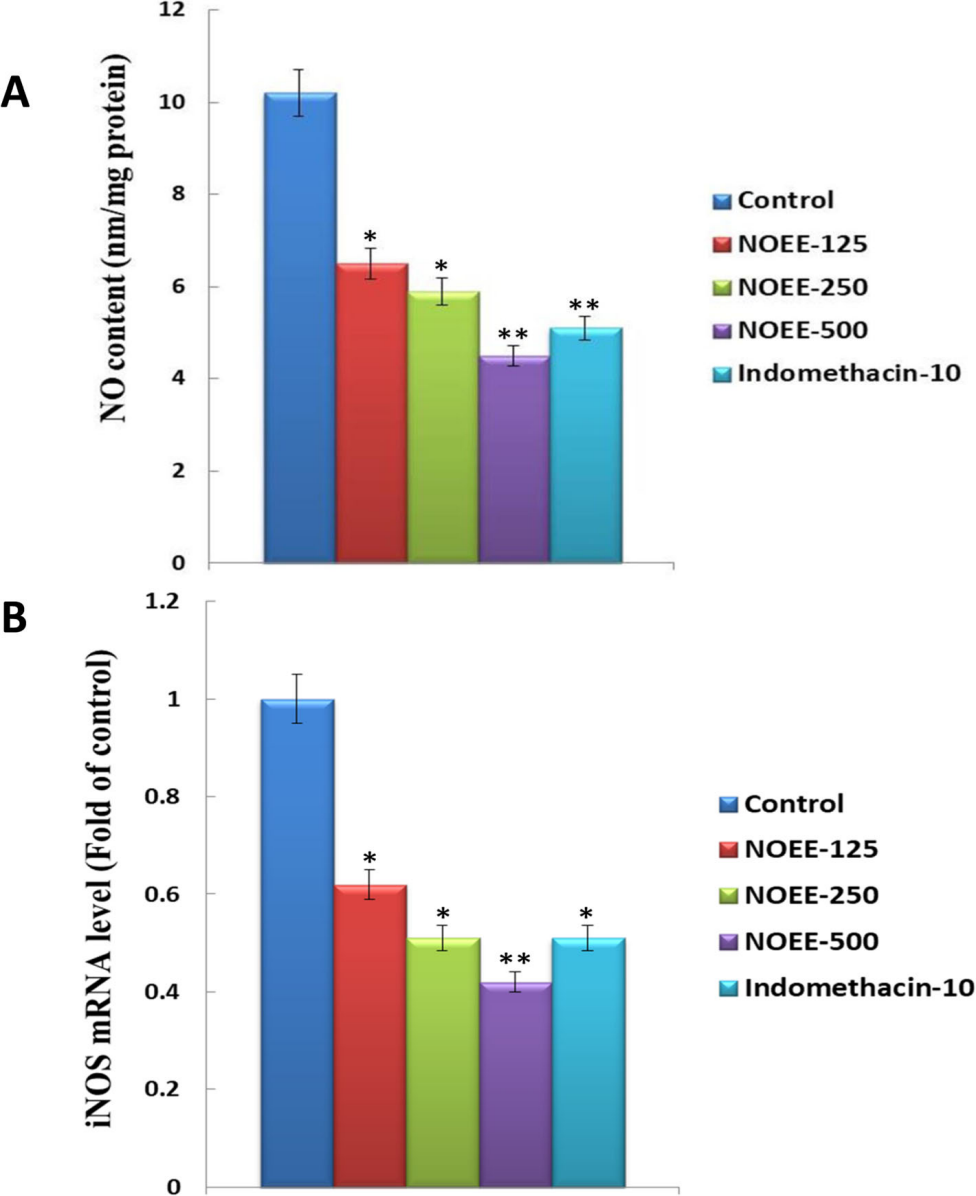
Effects of NOEE on **a** NO production **b** and mRNA expression of iNOS in rat paw of carrageenan-treated rats. Values are given as x̄ ± S.E.M. (*n* = 10). *p ≤ 0.05 significant and **p ≤ 0.005 are highly significant as compared to control. However Naïve = 0.0 for both **a** and **b**

Figure 6a shows the activity of NOEE on the production of NO while Fig. 6b presented the iNOS mRNA expressions in the carrageenan-induced rat paw edema. At 250 mg/kg and 500 mg/kg doses of plant extract, a significant and highly significant reduction was observed in the production of NO by 42.1 and 55.8% respectively compared to the control. Similarly, at the doses of 125, 250 and 500 mg/kg NOEE, iNOS mRNA expression was also suppressed significantly in dose dependent manner by 38.1, 49.5 and 58.3% respectively. However, standard indomethacin also attenuated the NO production (50.1%) and iNOS (49.6%) mRNA expressions in a significant manner in comparison to the control group.

#### Effects of NOEE on the production of PGE_2_ and mRNA expression of COX-2 in carrageenan-induced rat paw edema

**Fig. 7:**
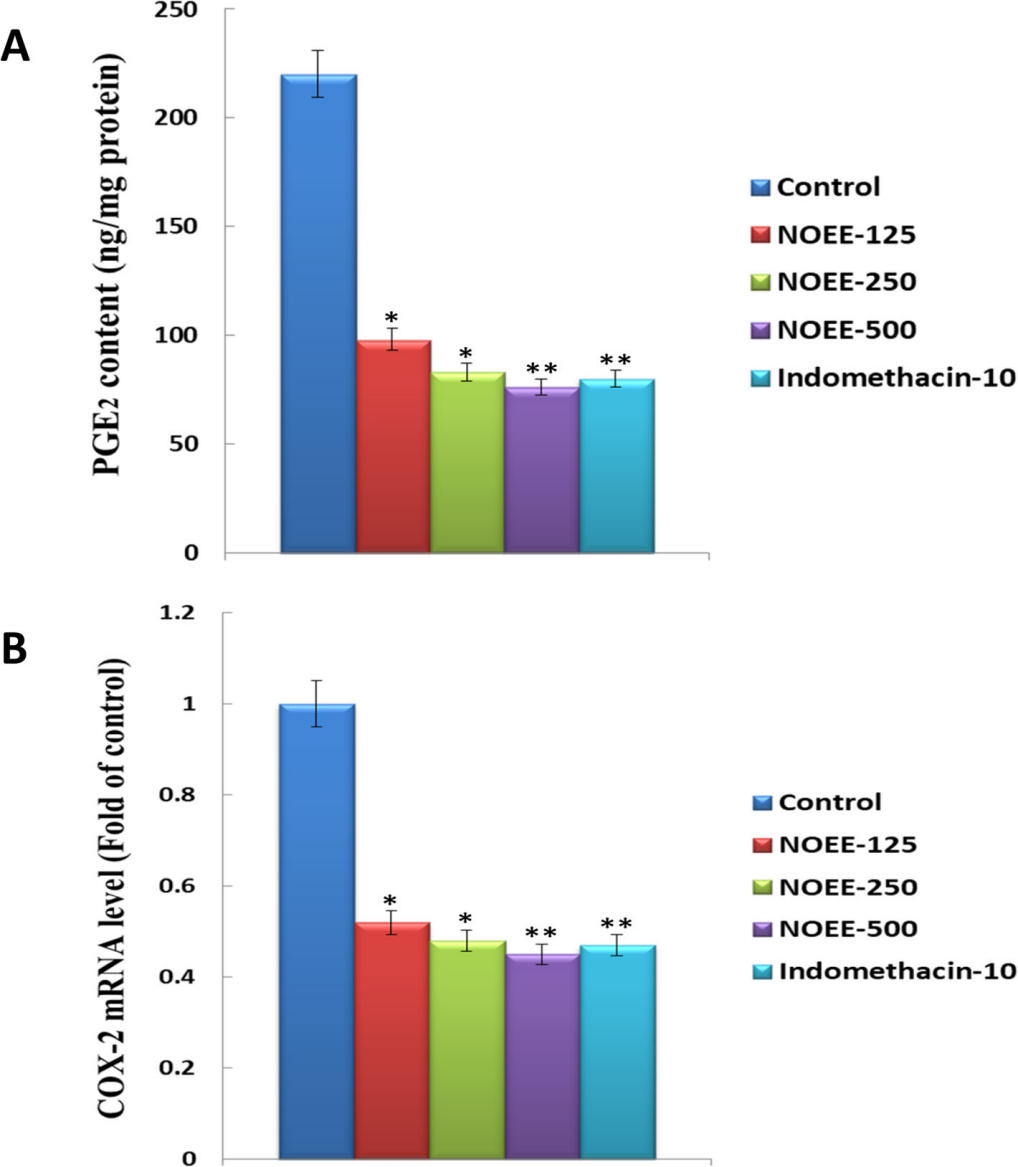
Effects of NOEE on **a** PGE_2_ production and **b** mRNA expression of COX-2 in rat paw of carrageenan-treated rats. Values are given as x̄ ± S.E.M. (*n* = 10). *p ≤ 0.05 significant and **p ≤ 0.005 are highly significant as compared to control. However Naïve group = 0.0 for both **a** and **b**

Figure 7a and b present the activity of NOEE on the production of PGE_2_ and mRNA expression of COX-2 respectively in the carrageenan-induced rat paw edema. NOEE at the dose of 125 and 250 mg/kg, significantly reduced PGE_2_ production by 55.4 and 62.2% respectively while highly significant attenuation (65.4%) was observed at the dose of 500 mg/kg as compared to the control group. NOEE reduced the COX-2 mRNA expression in the ranges of 36.2 to 55.8% while a highly significant effect was observed at the dose of 500 mg/kg i.e. 55.8%. Furthermore, the achieved outcomes were much comparable with a standard group for the reduction of PGE_2_ production and COX-2 mRNA expression by 63.6 and 50.3% respectively.

#### Effects of NOEE on the production of TNF-α and IL-1β and their mRNA expression in carrageenan-induced rat paw edema

**Fig. 8:**
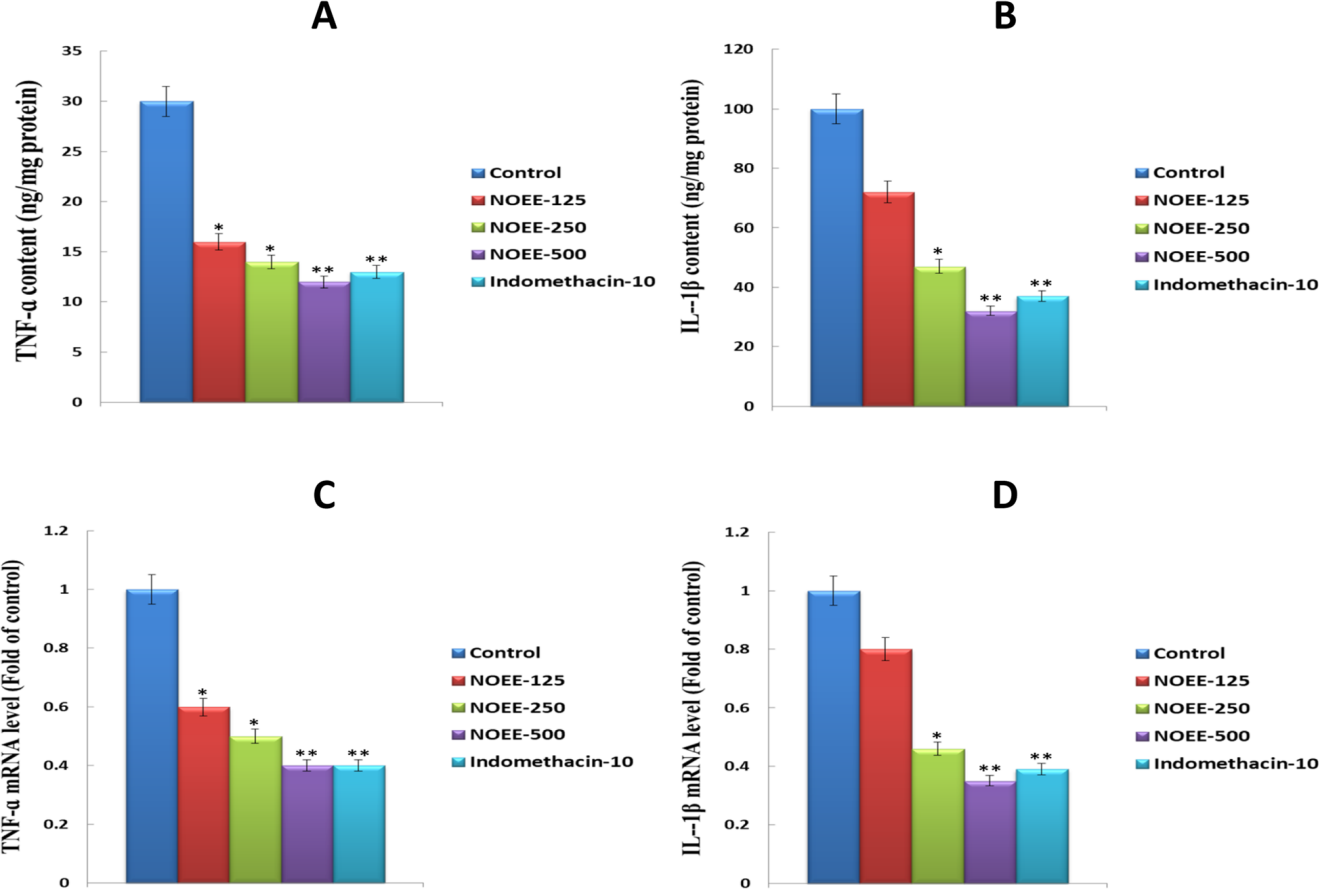
Effects of NOEE on the production of **a** TNF-ɑ and **b** IL-1β while **c** and **d** show mRNA expression of TNF-ɑ and IL-1β respectively in rat paw of carrageenan-treated rats. Values are given as x̄ ± S.E.M. (*n* = 10). *p ≤ 0.05 significant and **p ≤ 0.005 are highly significant as compared to control. However Naïve group = 0.0

The effects of different concentrations of NOEE on TNF-α and IL-1β levels in carrageenan-induced rat paw edema are presented in Fig. 8a and b respectively. The dose-dependent suppressions in TNF-α and IL-1β levels were observed in the ranges of 46.6 to 60.1% and 28.3 to 68.4% respectively after treatment with NOEE at different doses. At 250 and 500 mg/kg doses of NOEE, significant and highly significant down-regulation in mRNA expressions of TNF-α compared with the control i.e. 50.2 and 60.7% respectively (Fig. 8c). Consistent with the findings of IL-1β levels, Fig. 8d also shows that NOEE remarkably reduced the mRNA expression of IL-1β in a dose-dependent manner. In addition, indomethacin at 10 mg/kg showed very similar effects to that of NOEE at 500 mg/kg.

#### Determination of inflammatory biomarkers

**Fig. 9:**
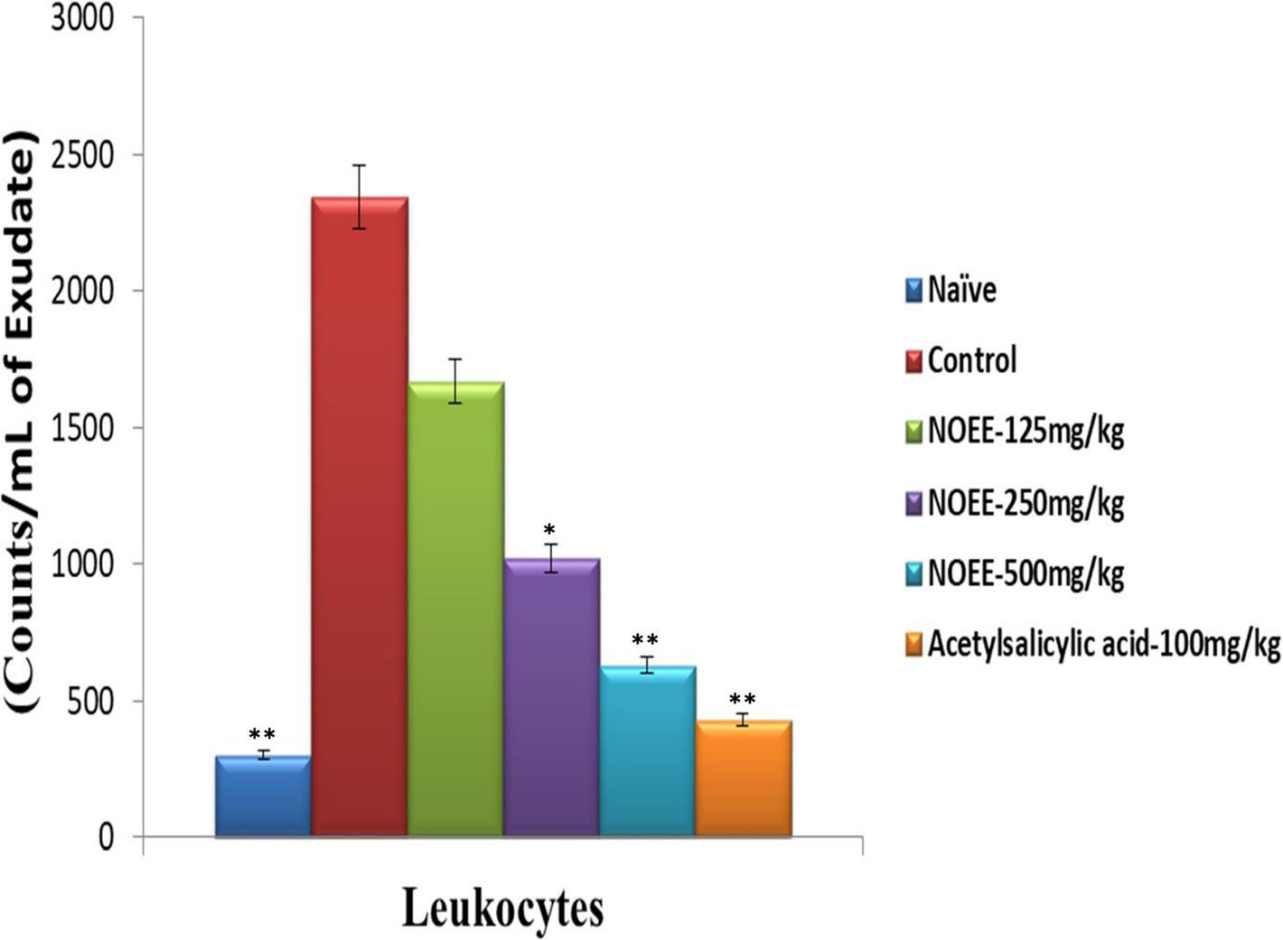
Effect of NOEE leukocyte counts in peritoneal inflammatory exudates. Values are given as x̄ ± S.E.M. (*n* = 10), *p ≤ 0.05 and **p ≤ 0.005 significant and highly significant as compared to control

**Fig. 10:**
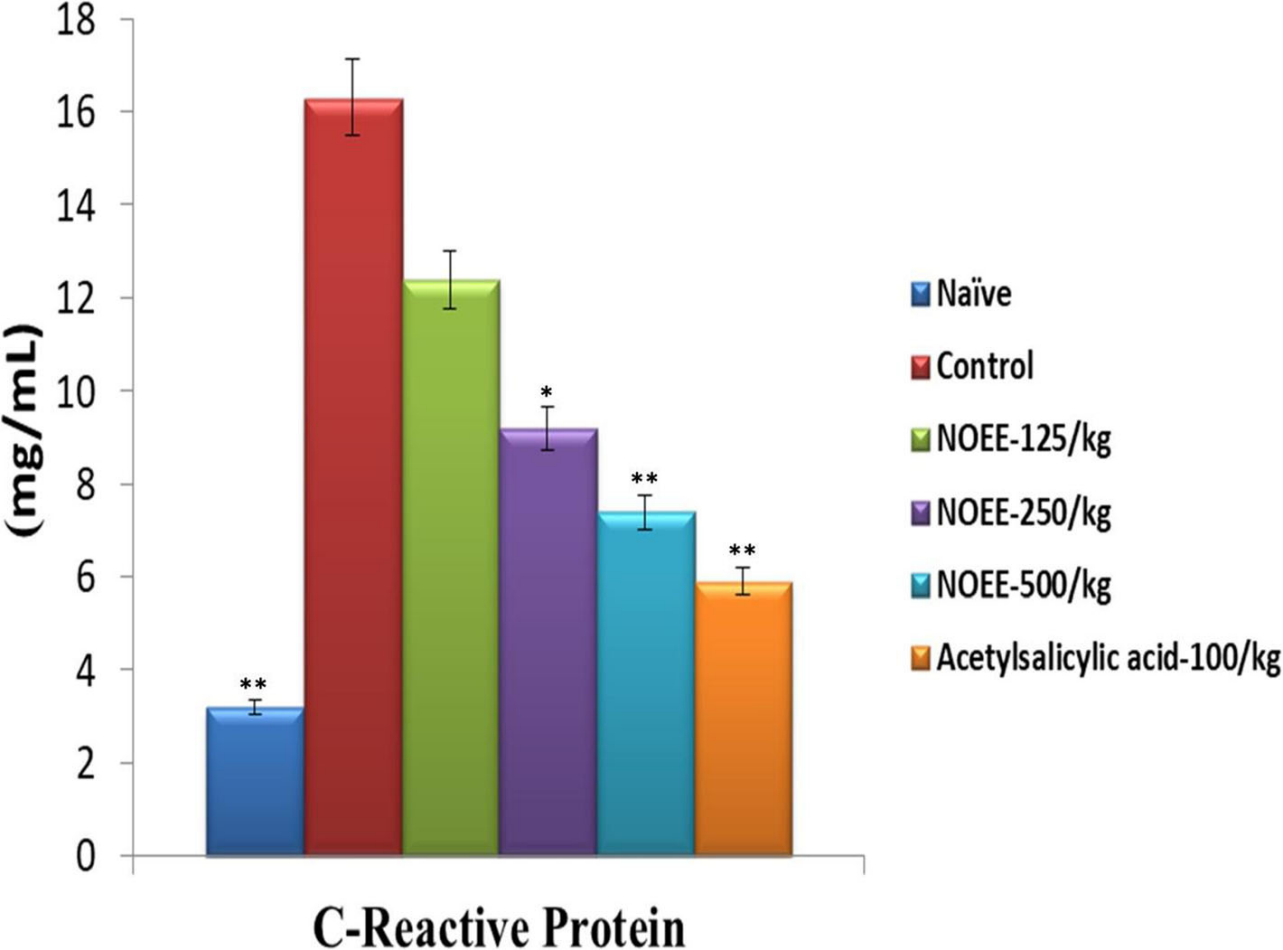
Effect of NOEE C-reactive protein levels in peritoneal inflammatory exudates. Values are given as x̄ ± S.E.M. (*n* = 10), *p ≤ 0.05 and **p ≤ 0.005 significant and highly significant as compared to control

The reduction in total leukocyte count and CRP levels by NOEE in peritoneal exudate after acetic acid-induced peritoneal inflammation are shown in Figs. 9, and 10 respectively. At 250 mg/kg and 500 mg/kg doses of plant extract, significant (*P < 0.05*) and highly significant (*P < 0.005*) reductions were observed in total leukocyte count and CRP levels. Highly significant reduction in total leukocyte count (73.09%) and CRP levels (54.60%) were found after treatment with NOEE at 500 mg/kg. However, the percentage reduction of extract was much lesser than acetylsalicylic acid at the dose of 10 mg/kg i.e. 81.63 and 63.80% reduction in total leukocyte count and CRP levels respectively.

**Fig. 11:**
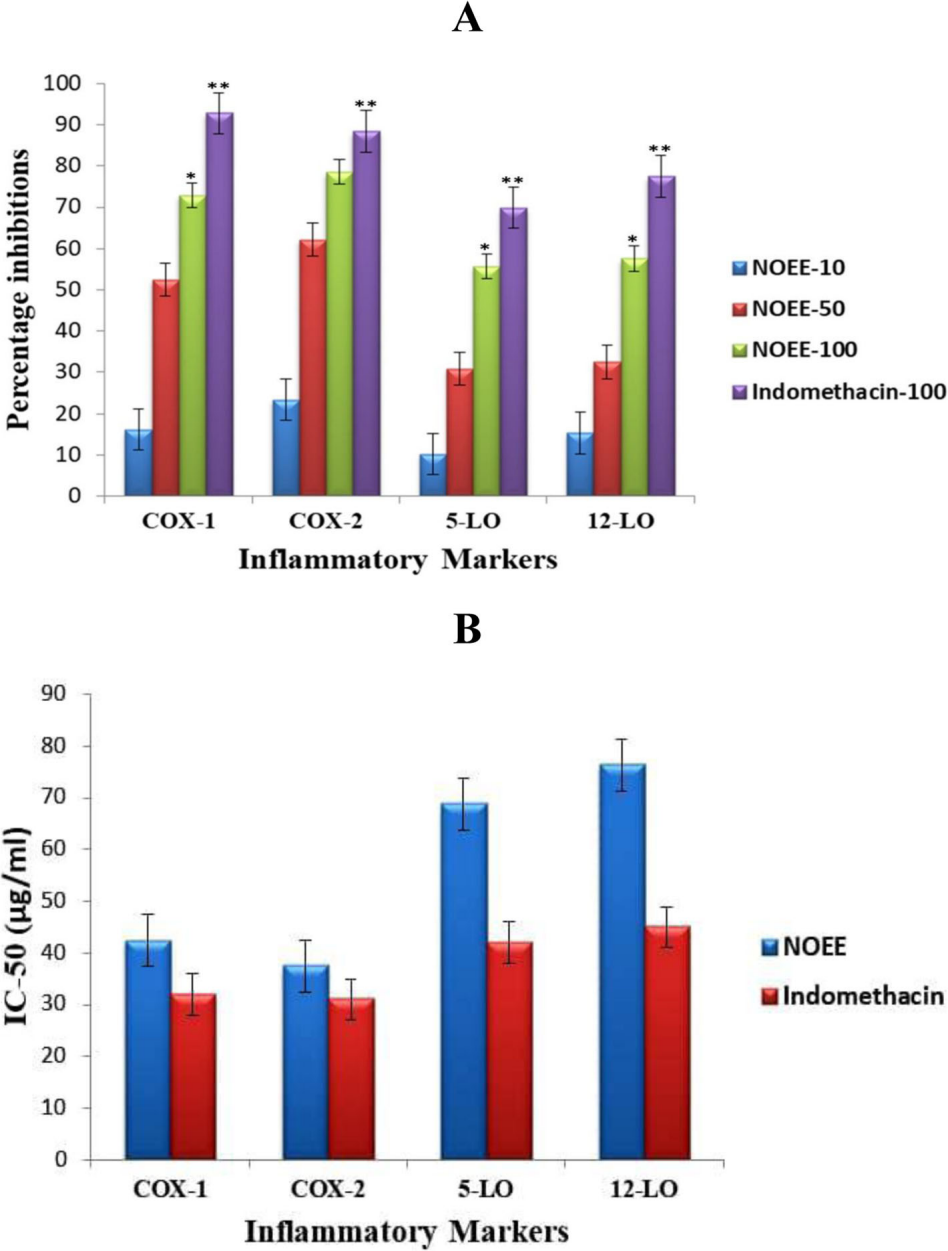
**a** Inhibition of COX-1, COX-2, 5-LO and 12-LO by NOEE **b** IC_50_ values of NOEE and indomethacin on COX-1, COX-2, 5-LO and 12-LO. Values are given as x̄ ± S.E.M. (*n* = 10) *p ≤ 0.05 significant as compared to control, **p ≤ 0.005 highly significant as compared to control

The percentage inhibition of COX-1, COX-2, 5-LO and 12-LO by NOEE at the concentrations of 10, 50 and 100 μg/mL are presented in Fig. 11a. NOEE demonstrated COX-1 inhibition in dose dependent manner with the percentage inhibitions of 16.1, 52.4 and 72.9% at the doses of 10, 50 and 100 μg/mL respectively. However, inhibition of COX-2 by NOEE was much greater than COX-1 inhibition. Similarly, NOEE exhibited marginally potent 12-LO inhibition (57.6%) compared to 5-LO inhibition (55.7%) at the concentration of 100 μg/mL. Moreover, the IC_50_ value of NOEE was much comparable with the indomethacin (standard) on COX-1, COX-2, 5-LO and 12-LO as presented in Fig. 11b. In addition, NOEE showed lower IC_50_ value for COX-1 and COX-2 (42.4 μg/mL and 37.5 μg/mL) than 5-LO and 12-LO (68.8 μg/mL and 76.4 μg/mL).

### In vivo acute and sub- toxicity studies

#### Clinical examinations, body weight and relative organ weights

Initially, the acute toxicity of NOEE was studied in rats for 14 days. No significant sign of toxicity was observed in tested rats after 14 days of oral administration of NOEE. The LD_50_ value of NOEE was found to be > 5000 mg/kg. In the sub-acute toxicity study, Table S2 shown that no significant changes in the body and relative organ weights were observed in both gender tested groups compared to the control group (*P > 0.05*). All tested animals had survived with no sign of abnormalities during the 28 days study period.

#### Hematological and biochemical analysis

After administration of NOEE, there were no significant changes found in the blood profile of studied groups in both male and female groups of animals as compared to control **(**Table 2**)**. Moreover, no abnormalities were observed in serum levels of sodium, potassium and calcium during sub-acute toxicity studies. At three different doses of NOEE, a significant increase of alkaline phosphatase (ALP), aspartate transaminase (AST), alanine transaminase (ALT) and bilirubin levels were observed at the dose of 2000 mg/kg in both genders groups compared to control groups. In contrast, the normal levels of creatinine, blood urea nitrogen (BUN) and uric acid were observed at all doses of NOEE.

**Table 2:** Effects of different concentration of NOEE on serum electrolyte levels, blood profile and liver profile in Sprague Dawley rats

Parameters	Groups* (mean ± S.D)							
	Male				Female			
	Control	NOEE (500 mg/kg)	NOEE (1000 mg/kg)	NOEE (2000 mg/kg)	Control	NOEE (500 mg/kg)	NOEE (1000 mg/kg)	NOEE (2000 mg/kg)
**Serum electrolytes**								
Na^+^ (mEq/L)	132.1 ± 5.21	137.3 ± 7.52	136.2 ± 7.46	134.6 ± 8.57	134.5 ± 5.71	138.2 ± 7.03	135.7 ± 7.81	136.2 ± 9.72
K^+^ (mEq/L)	5.26 ± 0.57	5.64 ± 0.48	5.69 ± 1.56	5.73 ± 0.98	5.87 ± 0.89	5.63 ± 0.73	5.79 ± 0.42	5.92 ± 0.67
Ca^+^ (mEq/L)	5.10 ± 0.52	5.36 ± 0.52	5.18 ± 1.28	5.23 ± 0.92	5.16 ± 0.73	5.21 ± 0.70	5.41 ± 0.67	5.25 ± 0.31
**Blood profile**								
RBCs (10^12^/L)	8.38 ± 0.42	8.26 ± 0.78	8.31 ± 0.32	8.12 ± 0.47	7.89 ± 0.93	7.83 ± 0.46	7.67 ± 0.92	7.92 ± 0.58
WBCs (10^9^/L)	13,351 ± 5242	13,831 ± 3170	13,687 ± 5904	14,073 ± 5340	11,920 ± 5027	11,804 ± 3737	12,034 ± 4329	11,974 ± 4542
Hb levels (g/dL)	14.23 ± 1.04	14.19 ± 0.75	14.71 ± 0.68	14.45 ± 0.72	13.06 ± 0.82	13.71 ± 0.27	13.43 ± 0.52	13.74 ± 0.63
ESR (mm/h)	1.41 ± 0.21	1.39 ± 0.23	1.34 ± 0.52	1.56 ± 0.42	1.49 ± 0.23	1.41 ± 0.47	1.47 ± 0.76	1.42 ± 0.53
**Liver profile**								
ALP (U/L)	209.47 ± 25.7	225.41 ± 77.6	219.56 ± 54.0	257.65 ± 56.5*	228.60 ± 56.3	241.38 ± 59.3	257.19 ± 27.6	270.41 ± 38.3*
AST (U/L)	233.72 ± 39.4	242.40 ± 27.1	278.37 ± 36.8	289.56 ± 57.5*	212.49 ± 44.3	209.37 ± 57.6	236.98 ± 38.2	261.09 ± 26.1*
ALT (U/L)	41.21 ± 14.27	45.79 ± 11.08	42.13 ± 6.25	46.19 ± 5.43*	51.10 ± 14.31	54.08 ± 9.40	58.97 ± 8.73	71.37 ± 9.33*
Bilirubin (mg/dL)	0.72 ± 0.21	0.61 ± 0.27	0.67 ± 0.13	0.73 ± 0.22*	0.54 ± 0.14	0.52 ± 0.12	0.63 ± 0.37	0.72 ± 0.29*
**Kidney profile**								
Creatinine (mg/dL)	1.82 ± 0.44	1.84 ± 0.24	1.78 ± 0.31	1.80 ± 0.17	1.10 ± 0.24	1.05 ± 0.42	1.23 ± 0.19	1.10 ± 0.15
BUN (mg/dL)	26.30 ± 3.21	24.51 ± 2.87	24.05 ± 4.18	22.96 ± 3.84	25.27 ± 2.89	26.97 ± 3.72	26.33 ± 2.94	25.94 ± 4.68
Uric acid (mg/dL)	1.73 ± 0.24	1.75 ± 0.18	1.68 ± 0.23	1.72 ± 0.15	1.64 ± 0.28	1.67 ± 0.20	1.72 ± 0.12	1.64 ± 0.34

*NOEE Nerium oleander* ethanolic extract, *RBCs* Red blood cells, *WBCs* White blood cells, *Hb* Hemoglobin, *ESR* Erythrocyte sedimentation rate, *ALP* Alkaline phosphatase;

*AST* Alkaline phosphatase, *ALT* Alanine transaminase, *BUN* Blood urea nitrogen

*p ≤ 0.05 significant as compared to control

## Discussion

In order to assess the antimicrobial potential of ethanolic flower extract of *N. oleander*, different concentrations were used against various highly resistant Gram-positive and Gram-negative clinical isolates. An antibiotic susceptibility test was also performed against clinical isolates. The extract showed significant results against *MRSA* and *S. epidermidis* in dose dependent manner followed by *S. aureus* and *S. enterica*. The extract is found to be highly sensitive against *S. aureus* at the lowest dose of 10 mg/mL. This organism is usually involved in skin infections and lesions and many antibiotics were already reported for their resistance pattern against this organism [40]. Test compound showed more powerful results against Gram-positive isolates compares to Gram-negative. Amoxicillin and gentamycin were found to be the most resistant antibiotics against most resistant clinical isolates. However, the statistical analysis reflects significant antibacterial potential between commercial antibiotics and experimental herbal extract. Obtained results are also in agreement with Namian et al. who investigated the antibacterial potential of flower extract of this plant in crude as well as in fraction form against 3 gram-negative and 3 gram-positive bacterial strains [41]. A crude extract was found to be more effective than purified fraction with significant results. The strong outcomes of the present study might be due to the selection of ethanol as extracting solvent since the importance of solvent extraction in obtaining powerful antimicrobial action has already been reported [42].

At present, limited data are available on time-killing kinetics of antimicrobials obtained by their MICs. In addition our study observed that NOEE shows inhibition in bacterial growth as well as organism proliferation at the same concentration against few tested strains. Due to this fact, time-killing assay was performed to acquire the relationship between incubation time, the concentration of antimicrobial agent and the rate of bactericidal activity [22]. NOEE showed significant bactericidal and bacteriostatic effects after short exposure with the tested isolates. It was observed that the extract produced bactericidal effects against most of the tested isolates within 2 h. The findings of the time killing assay showed that the extract produced their bactericidal effects more rapidly against Gram-negative organisms.

Previously several studies reported the bacterial killing mechanism of different antibacterial agents using the SEM technique [43, 44]. The present study findings demonstrated that the extract was highly susceptible against *MRSA* among all other tested isolates. Therefore, SEM analysis was carried out on *MRSA* cells by examined the morphological changes in cells after exposure with NOEE to illustrate the possible bacterial killing mechanism of NOEE. The significant morphological and cytological alterations in bacterial cell membrane were observed and cells were completely lysed and collapsed after 12 h treatment with NOEE. This could be suggested that NOEE had a high binding affinity with the bacterial cell membrane lipopolysaccharides*.* This extract-lipopolysaccharide interaction significantly altered the morphology of the bacterial cell membrane [45]. Similar alterations in the morphology of bacterial cells were reported in previous studies after treatment with different plant extracts at their MICs. For instance, Shafiq et al., and Mumtaz et al. observed much identical rapturing in the membrane of *MRSA* and *S. pneumonia* cells after treated with *Casuarina equisetifolia* and *Sphaeranthus indicus* respectively [9, 28].

To evaluate the anti-inflammatory effects of plant extract, it is very important to find the extract effectiveness in both early and late phases of inflammation. Here two well-established animal models were used to investigate the anti-inflammatory activity of NOEE. The granuloma induced by cotton pellet is a commonly reported method in animal studies for the evaluations of late phase (chronic) anti-inflammatory potential of testing a drug or any other anti-inflammatory agents. The cotton pellet implantation in the subcutaneous region is directly responsible for the activation of acute inflammation. Later, this acute inflammation eventually leads to a chronic phase [36]. NOEE showed significant inhibition of cotton pellet induced granuloma formation at doses of 125, 250 and 500 mg/kg. Hence, the data of this model suggested that the NOEE has significant potential to produced anti-inflammatory activity at the chronic phase of inflammation.

To screen the anti-inflammatory activity of the test solution, carrageenan-induced acute inflammation is supposed to be the most suitable test. In this model, edema development is considered as a biphasic curve since the inflammation occurs in 1st h either by trauma injection or due to the release of histamine and serotonin [32]**.** However, it was observed that no reduction in edema in the early hours at all doses of NOEE. Although in the second phase of inflammation (3rd h), a considerable reduction in paw edema was noticed at the dose of 500 mg/kg. Our results are in streamline with the study of Erdemoglu et al. who reported the significant anti-inflammatory activity of different plants belongs to the *Apocynaceae* family [46]. The inhibitory effects of the experimental extract might be observed due to the inhibition of the cyclooxygenase pathway, which leads to the inhibition of prostaglandins [36].

The involvement of COX-2 expression in the progression of inflammatory pathogenesis was observed in various diseases such as central nervous systems, gastrointestinal tract, ischemia and inflammation of the lung and liver [47, 48]. In the present study, NOEE reduced PGE_2_ production and COX-2 mRNA expression in carrageenan-induced rat paw edema in a dose-dependent manner, indicating that NOEE produced anti-inflammatory activity by inhibiting the expression of COX-2 mRNA level.

TNF-α and IL-1β are the highly substantial inflammatory mediators reported in association with several inflammatory diseases including rheumatoid arthritis, bacterial sepsis, skin inflammation and many others [49, 50]. At an initial inflammatory stage, the release of these pro-inflammatory cytokines is observed in response to various inflammatory stimuli resulting in the increased production of inflammatory mediators such as prostaglandins and NO [51]. Furthermore, TNF-α and IL-1β are also responsible for the inflammatory responses involved in carrageenan-induced inflammation [52]. It is reported that TNF-α mediates the production of IL-1β, which causes the release of cyclooxygenase enzyme [53]. However, the oxidation L-arginine nitrogen atom by the enzyme nitric oxide synthase (NOS) is responsible for the generation of NO. This NO is also contributing to the inflammatory responses, produced in carrageenan-induced inflammation [54]. Similarly, it is also found that NO promotes the production of prostaglandins in carrageenan-induced edema [55]. Hence, it is an effective strategy to suppress these cytokines for the management of different types of inflammatory disorders. In the present study, NOEE normalized the levels of TNF-α, IL-1β and iNOS and their mRNA expressions in a dose-dependent manner which were increased after carrageenan treatment in the rat paw. It is suggested that the anti-inflammatory activity of NOEE is also mediated by the inhibition of TNF-α, IL-1β and iNOS mRNA expressions.

It has been reported that different types of acute inflammations result in the release of different inflammatory mediators in peritoneal fluids such as CRP, leukocytes COX-1, COX-2, 5-LO and 12-LO [56]. These chemicals sensitize and activate peripheral chemical mediated inflammatory responses. Basically, these types of responses generally occur due to the occurrence of peripheral inflammation [57]. The experimental extract showed a significant decrease in total leukocyte count and CRP levels at the dose of 500 mg/kg. It seems that this reduction in CRP and leukocyte levels also provided us substantial evidence that NOEE has a significant potential to produce anti-inflammatory activity.

In the current research, we found that experimental extract having an inhibitory effect on both COX-1 and COX-2 enzymes in dose dependent manner. Interestingly, it was observed that extract showed a more potent inhibitory effect on COX-2 compared to COX-1 enzymes. The selective COX-2 inhibitors show therapeutic activity with few ulcers toxicity in GI tracts. A parallel relationship was suggested among COX-2 selectivity and NSAID treatment-related GI side effects since the COX-2 selective compounds showed fewer ulcerative effects [58]. Similarly, lipoxygenase perform an imperative part in the pathophysiology of numerous inflammatory diseases [59]. Enzymes belong to the LOX group are responsible for catalyzing deoxygenation of polyunsaturated fatty acids. Present study results reflect the promising anti-LOX effects of NOEE; this might be due to the antioxidant potential of this plant.

In addition, no significant sign of toxicity was observed in Sprague Dawley rats after 14 days oral dosing of NOEE. A sub-acute toxicity study for 28 days was also performed to evaluate the toxic potential of plant extract. No significant changes were observed in body weights and relative organ weights of both genders at any dose of plant extract compared to control groups. All studied animals survived during the whole toxicity study until euthanasia was performed for further studies. No significant changes were observed in serum sodium, potassium and calcium levels and hematological parameters of studied animals after 28 days oral dosing of plant extract.

The biochemical analyses of blood serum are a widely used tool to assess the responses in living beings induced by different exogenous chemicals. The elevated levels indicate any damage or toxicity of body organs. After 28 days exposure to plant extract at three different doses, a significant increase was observed in hepatic enzymes and bilirubin levels at the dose of 2000 mg/kg in animals of both genders. This increased in enzymatic levels indicated the chances of hepatocellular injury at higher doses of plant extract. In cholestatic drug reactions, these types of enzymatic elevations along with hyperbilirubinemia are also observed [39]. However, this mild hepatotoxicity was observed at the dose of 2000 mg/kg; which were about several times higher than the above defined effective concentrations for anti-inflammatory activity i.e. 100 mg/kg. In addition, the normal levels of creatinine, BUN and uric acid at all doses indicated that plant extract did not produce any toxic effects on renal systems.

However, an extract of *N. oleander* used in our study was not much similar in its solvent properties as to the traditional approach where the population used aqueous extracts while we have used ethanolic extract due to the greater pharmacological activity reported in previous studies. In addition, it will be more beneficial to conduct in vivo human studies on *N. oleander* extract and should determine pharmacokinetic properties, adverse effects and serum-attainable levels.

## Conclusion

In conclusion, this study demonstrated the significant dose-dependent anti-bacterial and anti-inflammatory activity of ethanolic flowers extract of *N. oleander* which could be exploited in the search for plant-based anti-inflammatory agents. The extract involved in the bacterial cell membrane alterations for anti-bacterial activity. However, the anti-inflammatory activity of plant extract was observed through the reduction in the levels and mRNA expressions of different pro-inflammatory mediators. In addition no toxicity was observed at therapeutics doses of plant extract in toxicity assay. Thus, the present study scientifically validated the traditional use of *N. oleander* flowers for the treatment of elevated pain and inflammation, particularly in bacterial infections.

## Supplementary Information

**Additional file 1: Table S1.** MIC and MBC of crude ethanolic flowers extract of *N. oleander.* **Table S2.** Body weight gain and relative organs weight of rats after treated with NOEE at different doses

## Data Availability

All data generated or analyzed during this study are included in this published article.

## Abbreviations

ALP: Alkaline phosphatase
AST: Aspartate transaminase
ALT: Alanine transaminase
BUN: Blood urea nitrogen
CRP: C-reactive protein
COX: Cyclooxygenase
CLSI: Clinical Laboratory and Standard Institute
ESR: Erythrocyte sedimentation rate
HETE: Hydroxyeicosatetraenoic acid
IL-1β: Interleukin-1β
LD: Lethal dose
LO: Lipoxygenase
MHA: Muller Hinton agar
MIC: Minimum inhibitory concentration
MBC: Minimum bactericidal concentration (MBC)
NO: Nitric oxide
NOEE: *Nerium oleander* ethanolic extract
NSAIDs: Non-steroidal anti-inflammatory drugs
NIH: National Institute of Health
ODs: Optical densities
PCSIR: Pakistan Council of Scientific and Industrial Research
PGE_2_: Prostaglandins-2
PI: Percent inhibitions
RBCs: Red blood cells
TSA: Tyramide Signal Amplification
TNF-α: Tumor necrosis factor-α
WHO: World health organization
WBCs: White blood cells
