# Supplementary material for: A mechanistic study on the inhibition of bacterial growth and inflammation by Nerium oleander extract with comprehensive in vivo safety profile
Source: BMC Complement Med Ther. 2021 May 1;21:135. doi: 10.1186/s12906-021-03308-z (PMC8088680; doi:10.1186/s12906-021-03308-z)
Supplement: Supplementary file 1 — Additional file 1: Table S1. MIC and MBC of crude ethanolic flowers extract of N. oleander. Table S2. Body weight gain and relative organs weight of rats after treated with NOEE at different doses [file 12906_2021_3308_MOESM1_ESM.docx]

**Table S1** MIC and MBC of crude ethanolic flowers extract of *N. oleander*

| **Clinical Isolates** | **MIC (mg/ml)** | **MBC (mg/ml)** |
| --- | --- | --- |
| ***S. aureus*** | 5.0 ± 0.62 | 5.5 ± 1.52 |
| ***B. subtilis*** | 6.5 ± 0.45 | 7.0 ± 0.71 |
| ***S. pyogenes*** | 8.5 ± 0.61 | 9.5 ± 1.40 |
| ***S. fecalis*** | 10.5 ± 0.80 | 10.5 ± 2.20 |
| ***S. epidermidis*** | 8.5 ± 0.54 | 9.5 ± 0.85 |
| ***S. enterica*** | 9.5 ± 1.20 | 10.0 ± 1.24 |
| ***MRSA*** | 8.0 ± 0.75 | 8.0 ± 1.40 |
| ***P. aeruginosa*** | 9.0 ± 0.66 | 10.0 ± 2.61 |
| ***K. pneumonia*** | 11.0 ± 1.47 | 12.0 ± 2.06 |
| ***S. typhi*** | 9.0 ± 2.70 | 10.0 ± 3.60 |
| ***E. coli*** | 8.0 ± 1.91 | 9.0 ± 2.42 |

Results are mean ± S.D of triplicate experiments

**Table S2** Body weight gain and relative organs weight of rats after treated with NOEE at different doses

| **Groups** | **Body weight gain**  **(After 28^th^ days)** | **Relative major organs weights (%)**  **(After 28^th^ days)** | | | | |
| --- | --- | --- | --- | --- | --- | --- |
|  |  | **Brain** | **Heart** | **Kidneys** | **Liver** | **Lungs** |
| **Male** | | | | | | |
| **Control** | 22.1 ± 3.2 | 0.57 ± 0.03 | 0.33 ± 0.03 | 1.10 ± 0.07 | 4.07 ± 0.35 | 0.42 ± 0.05 |
| **NOEE (500 mg/kg)** | 21.5 ± 3.4 | 0.56 ± 0.05 | 0.33 ± 0.04 | 1.08 ± 0.10 | 4.24 ± 0.24 | 0.39 ± 0.08 |
| **NOEE (1000 mg/kg)** | 17.4 ± 4.5 | 0.61 ± 0.03 | 0.32 ± 0.02 | 1.03 ± 0.05 | 4.31 ± 0.27 | 0.44 ± 0.11 |
| **NOEE (2000 mg/kg)** | 18.3 ± 6.2 | 0.58 ± 0.03 | 0.33 ± 0.04 | 1.09 ± 0.11 | 4.51 ± 0.14 | 0.37 ± 0.06 |
| **Female** | | | | | | |
| **Control** | 19.2 ± 6.2 | 0.57 ± 0.05 | 0.35 ± 0.04 | 1.12 ± 0.09 | 4.54 ± 0.14 | 0.56 ± 0.07 |
| **NOEE (500 mg/kg)** | 17.1 ± 6.4 | 0.53 ± 0.03 | 0.37 ± 0.02 | 1.04 ± 0.08 | 4.38 ± 0.16 | 0.49 ± 0.04 |
| **NOEE (1000 mg/kg)** | 19.4 ± 5.3 | 0.56 ± 0.06 | 0.33 ± 0.04 | 1.16 ± 0.07 | 4.43 ± 0.36 | 0.54 ± 0.05 |
| **NOEE (2000 mg/kg)** | 18.0 ± 5.5 | 0.52 ± 0.04 | 0.38 ± 0.04 | 1.05 ± 0.04 | 4.52 ± 0.24 | 0.48 ± 0.05 |

Values are given as x̄ ± S.E.M. (n = 10)
